# Vasomotor Rhinitis: Current Concepts and Emerging Therapies

**DOI:** 10.7759/cureus.95631

**Published:** 2025-10-28

**Authors:** Ghezlan Aldawas, Imtiyaz N Bhat

**Affiliations:** 1 Otolaryngology, Head and Neck Surgery, Farwaniya Hospital, Kuwait City, KWT; 2 Otolaryngology, Head and Neck Surgery, Ministry of Health, Kuwait City, KWT

**Keywords:** botulinum toxin, capsaicin, cryotherapy, idiopathic rhinitis, intranasal ipratropium, non-allergic rhinitis, posterior nasal nerve ablation, rhinorrhea, vasomotor rhinitis, vidian neurectomy

## Abstract

Vasomotor rhinitis (VMR) is a non-infectious, non-allergic subtype of rhinitis, which is characterized by nasal blockage, liquid runny rhinorrhea, and increased sensitivity to nonspecific nasal triggers (such as changing temperature and smell). Although VMR is a disease with a high prevalence rate, it is wrongly diagnosed and under-treated due to its clinical manifestation with allergic rhinitis. Databases such as PubMed, EMBASE, and Cochrane Library were searched to obtain articles published between 2000 and 2025. Randomized controlled trials, cohort studies, and major clinical guidelines were included, while case reports and non-English studies were excluded to ensure consistency and reproducibility of the findings. Existing evidence shows that multiple regulations of the autonomic nervous system, neuropathway neuroplasticity and inflammation, and excessive quantitative stimulation of transient receptor potential (TRP) channels all play a role in the pathophysiology of VMR. Variable therapies include corticosteroids, intranasal antihistamines, and anticholinergics, although ipratropium bromide has had the best dependability results in terms of rhinorrhea. Surgical procedures that may be considered include intranasal capsaicin, botulinum toxin injections, ablation of the posterior nasal nerve (radiofrequency ablation or cryotherapy), and vidian neurectomy, but these interventions are not yet conclusively demonstrated or established as promising to help the patient with refractory symptoms. VMR is a condition that has not been adequately studied but is common and requires a progressive management style that relies on triggers. Whereas conventional intranasal treatment is the most appropriate in the first case, nerve-specific treatment has promising outcomes in the case of relapse.

## Introduction and background

Vasomotor rhinitis (VMR) refers to a chronic, mild, non-allergic or environmental type of rhinitis that is linked to nasal congestion, rhinorrhea, and response to non-specific or environmental stimuli [1]. In comparison to allergic rhinitis, which implies the participation of IgE-mediated immune responses [2], VMR is triggered by neurogenic and autonomic dysfunction contributing to the increase in nasal hyperreactivity [3]. It is a subtype of non-allergic rhinitis (NAR) [4], which is a heterogeneous collection of disorders with rhinitis symptoms neither due to atopy nor to infection, and which are sometimes lumped with other phenotypes like the gustatory, hormonal, or drug-induced rhinitis [5]. 

Recent literature has also introduced the broader term *Non-Allergic Rhinopathy* (NAR) to encompass VMR and other non-IgE-mediated nasal hyperreactivity disorders, reflecting the evolving understanding and updated classification of these conditions in recent consensus reports [3].

VMR represents a notable global health burden, with prevalence rates ranging from 5% to 25% depending on the studied population and geographic region [5]. Epidemiological data suggest slight regional and climatic variations, with a roughly equal distribution between males and females and symptoms more frequently reported in adults [5]. The severity of the disease is commonly assessed using standardized tools such as the reflective Total Nasal Symptom Score (rTNSS), Sino-Nasal Outcome Test (SNOT-22), and visual analog scales (VAS), which quantify symptom burden and treatment outcomes [6].

VMR is a clinically significant disease as it has a very high prevalence rate and affects the quality of life [1]. Common symptoms in patients include symptoms of chronic nasal congestion, rhinorrhea, and postnasal drip that influence sleep, daytime productivity, and cause fatigue and social discomfort [1]. The literature available points out the need to have a practical and evidence-based framework to manage VMR. Even though the use of such conventional agents as intranasal antihistamines, corticosteroids, and ipratropium yields moderate relief, a considerable number of patients continue to experience symptoms, and that is why new interventional options were created.

## Review

Methods

We conducted a narrative review and a targeted search of PubMed, EMBASE, and Cochrane Library databases for studies published between January 2000 and March 2025 to obtain up-to-date literature on VMR. The keywords used in the search strategy were VMR, nonallergic rhinitis, and idiopathic rhinitis in combination with the names of the sub-keywords’ pathophysiology, diagnosis, treatment, therapy, capsaicin, ipratropium, botulinum, posterior nasal nerve (PNN), vidian, cryo, and radiofrequency. The criterion for the eligible publications comprised randomized controlled trials, cohort studies, case series, and major guidelines or practice parameters pertinent to VMR [7,8]. The articles were limited to English human studies. Studies in which a different subgroup of VMR (i.e., applied to cases of allergic rhinitis, infectious rhinitis, or chronic rhinosinusitis with polyps) was not indicated were excluded.

Patient-reported symptom scores, including the reflective Total Nasal Symptom Score (rTNSS), Sino-Nasal Outcome Test (SNOT-22), and visual analog scale (VAS), were the primary outcomes of interest, along with the prevalence of rhinorrhea, use of rescue medication, and safety or adverse event profiles. Given the heterogeneity of the study design and endpoints, data were synthesized descriptively. This methodology enabled the incorporation of mechanistic understanding and clinician outcomes to present a clinically based summary of the prevailing concepts and emerging therapies in VMR [9].

To ensure methodological rigor and reproducibility, randomized controlled trials, cohort studies, and high-quality clinical guidelines were prioritized. Case reports, non-English studies, and publications with unclear diagnostic criteria were excluded to maintain consistency and evidence reliability.

Pathophysiology

The multifactorial nature of the pathophysiology of VMR is supported by recent research implying that VMR is caused mainly by the dysregulation of neural control in the nasal mucosa, not by IgE-mediated allergic responses. The most important feature of this process is the imbalance of the autonomic nervous system, i.e., parasympathetic hyperactivity, preconditioning glandular hypersecretion, and mucosal vasodilation. The result of this imbalance is sustained rhinorrhea and congestion devoid of evidence of inflammatory and infectious stimuli [9, 10]. Physiologic evidence, such as studies indicating autonomic dysfunction in VMR by authors who focus on abnormal response during stimulation of nasal secretory function in such patients, has supported the theory of parasympathetic predominance in such patients [10].

Other than autonomic imbalance, the hyper-reactivity of the nose is also contributed to by neuropeptides. Examples of molecules mediating neurogenic inflammation resulting in vasodilation, plasma extravasation, and the hyperreactivity of the mucosa include substance P, calcitonin gene-related peptide (CGRP), neurokinin A, and others [11]. These pathways explain exaggerated nasal response to non-inflammatory stimuli in VMR patients in a mechanistic manner.

TRP ion channels, especially TRPV1, are also involved in the pathologic sensory processing of VMR. Overexpression of TRP channels has been detected in patients with idiopathic rhinitis, which is associated with increased sensitivity to environmental stimuli (temperature, humidity, smoke, strong odors, alcohol, and cold air) [12,13]. An indirect indication that this pathway is important in rhinorrhea and nasal blockage symptoms comes in the form of capsaicin therapy, which desensitizes sensory fibers with TRPV1 expression [13,14].

Histologic examinations also indicate that there exists heterogeneity in the inflammatory patterns of cellular inflammation in VMR. Other patients have been known to display neutrophilic infiltration in the form of some form, or other patients will present an eosinophilic presentation, overlapping with NAR with eosinophilia syndrome (NARES). These differences may explain the inconsistent treatment responses observed across different patient groups [14].

Collectively, the new evidence contributes to an opinion that VMR should be seen as a disease of neurogenic hyperreactivity and should not be regarded as an IgE-based allergic process.

Takeaway: VMR is a neurogenic hyperreactivity disorder rather than an IgE-driven allergy.

Clinical features and diagnosis

Patients with VMR normally present with watery rhinorrhea, nasal congestion, sneezing, and postnasal drip. The symptoms tend to be perennial yet typically stimulated by nonspecific surroundings like the rise of temperature changes, strong smell, or alterations of humidity [6]. In contrast to allergic rhinitis, VMR is not related to pruritus or seasonal fluctuations, and the symptom is present in the presence of negative allergy test results. When examined, the nose mucosa is typically normal, but clear liquid secretions can be evident.

The diagnosis is based on exclusion. Allergic rhinitis is eliminated by negative skin-prick testing and serum IgE, whereas infectious rhinitis, chronic rhinosinusitis plus polyps, rhinitis medicamentosa, and other NAR subtypes, including gustatory or hormonal rhinitis, must be eliminated with meticulous history and endoscopic analysis [6]. Chronic causes, especially antihypertensives or hormonal therapies induced by anesthetic drugs, should also be taken into consideration. Standardized indications of symptom burden and the response to treatment are offered by outcome measures, such as reflective rTNSS, SNOT-22, and VAS [6,15]. Imaging or nasal endoscopy is only used where structural pathology or sinonasal disease is considered.

Management conventional therapies

Management of VMR is normally initiated by non-pharmacologic modalities that aim at the reduction of symptom triggers. Patients have been recommended to abstain from environmental stimuli that are known to accentuate symptoms, including abrupt shifts in temperature, exposure to perfumes, tobacco, alcohol, or other nonspecific irritants. Since such triggers occur very often and are often inescapable in everyday life, the adjunctive therapies are prioritized. As shown in Table 1, one of the safest and most widely recommended interventions is saline irrigation, which provides mechanical clearance of secretions and irritants, hydrates the nasal mucosa, and offers temporary relief of congestion [15].

**Table 1:** Conventional therapies in vasomotor rhinitis. Overview of first-line and pharmacologic treatment options, including mechanisms of action, primary symptom targets, supporting evidence, and other effects. Data compiled from [15-17].

Therapy	Mechanism of action	Main symptom controlled	Evidence level	Common side effects
Nasal antihistamines (e.g., azelastine)	Neural modulation; anti-inflammatory(mild) effects; lessening nasal hyperreactivity.	Sneezing, congestion, rhinorrhea.	Randomized controlled trials (RCTs); cohort studies	Bitter taste, sedation, nasal irritation
Intranasal corticosteroids(e.g., fluticasone, mometasone)	Anti-inflammatory; inhibition of mucosal edema and release of mediators.	Blockage, postnasal drip (variably useful rhinorrhea)	RCTs; evidence; mixed observations.	Dryness of the nose, epistaxis, and sore throat.
Intranasal anticholinergic(ipratropium bromide)	Glandular secretion (anti-secretory effect), parasympathetic inhibition.	Rhinorrhea, post-nasal drip, watery.	RCTs; guideline-supported	Nose dryness, slight irritation, and the occasional use of epistaxis.
Saline irrigation(isotonic or hypertonic)	Mechanical cleansing of mucus and irritants; hydration of the nasal mucosa	Congestion, postnasal drip, and general symptom relief	Observational studies; case series	Mild nose burning, short-lived irritation.

Pharmacologic treatment is resorted to when the conservative approach fails. Azelastine intranasal antihistamines are especially applicable in VMR where an allergic etiology is not observed. They are believed to exert their efficacy through neural modulation and a decrease in nasal hyperreactivity, but not through IgE-mediated pathways blockade. Their efficacy in enhancing rhinorrhea and congestion is clinically proven, and further benefits can be seen in their application in combination regimens [16].

Although intranasal corticosteroids are an important therapy for allergic rhinitis, they show inconsistent efficacy in VMR. Some studies, however, have suggested topical corticosteroids as a potential first-line option for non-allergic rhinopathy, though their overall effectiveness in VMR remains variable and inconsistent across clinical studies [16]. They may be most beneficial when underlying mucosal inflammation is present, but findings in the literature remain variable [16].

Intranasal anticholinergics, especially ipratropium bromide, have the most predictable benefit on watery rhinorrhea among the available agents. Their effect is on parasympathetic over-exertion, where they inhibit gland secretions such that they find significant application in patients who experience excess secretions of the nose. Guideline updates as well as randomized controlled trials identify ipratropium to be the main pharmacologic intervention in patients with VMR and noticeable rhinorrhea [17].

Combination therapy has become a viable alternative for patients with persistent or multifaceted symptoms. Antihistamine-containing corticosteroid combination sprays have demonstrated better effectiveness than monotherapy in certain cohorts, which is strongly suggestive of synergistic advantages due to the combined anti-inflammatory and neural effects [17].

Finally, the conventional therapies are the basis of VMR management. Individual patient-based treatment is essential, with periodic re-evaluation of symptom management and escalation when patients are still symptomatic following four to six weeks of best treatment. Patients who have not responded to medical management could be referred to more recent procedural interventions, although traditional agents are the preferred first-line modality because of accessibility, safety profile, and available evidence. Their weaknesses, however, project the necessity of new forms of treatment that would address the neurogenic processes that are behind this syndrome.

Emerging and procedural therapies

For patients who display VMR and do not respond to traditional medical therapy, many newer and procedural treatments have been investigated. These approaches target the neurogenic mechanism of the disorder more directly and have more clinical support.

One of the most researched novel interventions is intranasal capsaicin therapy, and its mechanism of action is desensitization of TRP channel-expressing sensory fibers. Short courses of capsaicin are of benefit in reducing nasal hyperreactivity and improving symptoms (particularly watery rhinorrhea). While the benefit is usually durable for weeks to months, application is associated with transient burning and nasal irritation that may limit tolerability [17].

Botulinum toxin injections into the inferior turbinate are another minimally invasive method [14]. By inhibiting cholinergic secretomotor activity, botulinum toxin decreases glandular output and has shown efficacy for rhinorrhea and congestion in case series and small randomized studies. The therapeutic effect generally lasts for three to six months, after which repeat injections may be required. The therapy is usually well tolerated, but local dryness and minimal discomfort may be observed.

More recently, PNN ablation, performed with radiofrequency or cryotherapy, has emerged as an office-based procedure with promising results. By selective intervention on postganglionic parasympathetic fibers, PNN ablation decreases both rhinorrhea and congestion, with minimal downtime. Adverse effects are usually mild and short-lived, such as crusted over skin, numbness, or momentary discomfort. Evidence supported by early prospective series [18].

In the past, in the treatment of severe refractory cases, vidian neurectomy was employed, which consisted of surgically sectioning the vidian nerve in the operating room. While effective, the use of this therapy has declined because of the risk of permanent complications, especially dry eye, and the availability of safer alternatives such as PNN ablation [19].

Finally, biologic agents and neuromodulation therapies are in the early stages of investigation, with a focus on targeting type 2 inflammatory pathways such as IL-4 and IL-13 blockade. Despite the encouraging nature of this data in other subtypes of rhinitis, their involvement in VMR remains rather unclear, with limited pilot data and no substantial randomized trials to date [19].

Collectively, these therapies give hope to refractory VMR, but with inconsistency in evidence generation, further high-quality studies are required to aid in how patients should be optimally selected, and the long-term consequences can be measured.

As shown in Table 2, emerging therapies for VMR include pharmacologic, procedural, and neuromodulatory options targeting neural pathways.

**Table 2:** Emerging therapies for vasomotor rhinitis. Overview of novel and interventional approaches targeting neural pathways, with details on mechanisms, settings, expected duration of benefit, evidence type, and adverse events. Data compiled from [14,17,18,19].

Therapy	Target/Mechanism	Setting	Duration of benefit	Evidence type	Advanced events
Capsaicin [17]	TRP channel desensitization of sensory fibers	Office/outpatient	Weeks-months	RCTs, pilot studies	Transient burning, nasal irritation
Botulinum toxin injection [14]	Cholinergic block of secretomotor activity	Office/outpatient	3-6 months	Case series, small RCTs	Local dryness, mild discomfort
Posterior nasal nerve ablation (radiofrequency/cryotherapy) [18]	Powdering down of post- or intra-glandular parasympathetic fiber.	Office/outpatient	1-2 years	Prospective series, early RCTs	Transient numbness, crusting, mild discomfort
Vidian neurectomy [19]	Surgical section of the vidian nerve	Operating room	Long-term	Historical data, case series	Dry eye, facial numbness, and increased surgical risk
Biologics (e.g., IL-4/IL-13 blockade) [19]	Instructing inhibition of cytokine pathways, neuromodulation.	Investigational	Unknown	Pilot studies, early data	Systemic effects, limited safety data

Practical treatment algorithm for VMR

The process of optimizing the management of VMR should be facilitated through a well-established stepwise progression. The initial step is proper phenotyping of VMR, ensuring that allergy testing is negative and that other causes, such as chronic rhinosinusitis or drug-induced rhinitis, are ruled out. Primary management revolves around the avoidance of triggers and the use of adjunctive saline irrigation. Pharmacologic therapy with intranasal ipratropium bromide is preferred for the treatment of rhinorrhea and is usually combined with an antihistamine or corticosteroid based on predominant symptoms. Capsaicin nasal therapy or botulinum toxin injections can be offered to patients experiencing persistent symptoms after four to six weeks, especially when rhinorrhea predominates. Patients who continue to have a considerable impact on quality of life despite optimal medical therapy are candidates for PNN ablation using radiofrequency or cryotherapy, which provides a longer duration of control. Vidian neurectomy remains a salvage procedure reserved for rare, refractory cases. Follow-up and treatment modification should be guided by standardized outcome measures such as rTNSS and SNOT-22.

**Figure 1:**
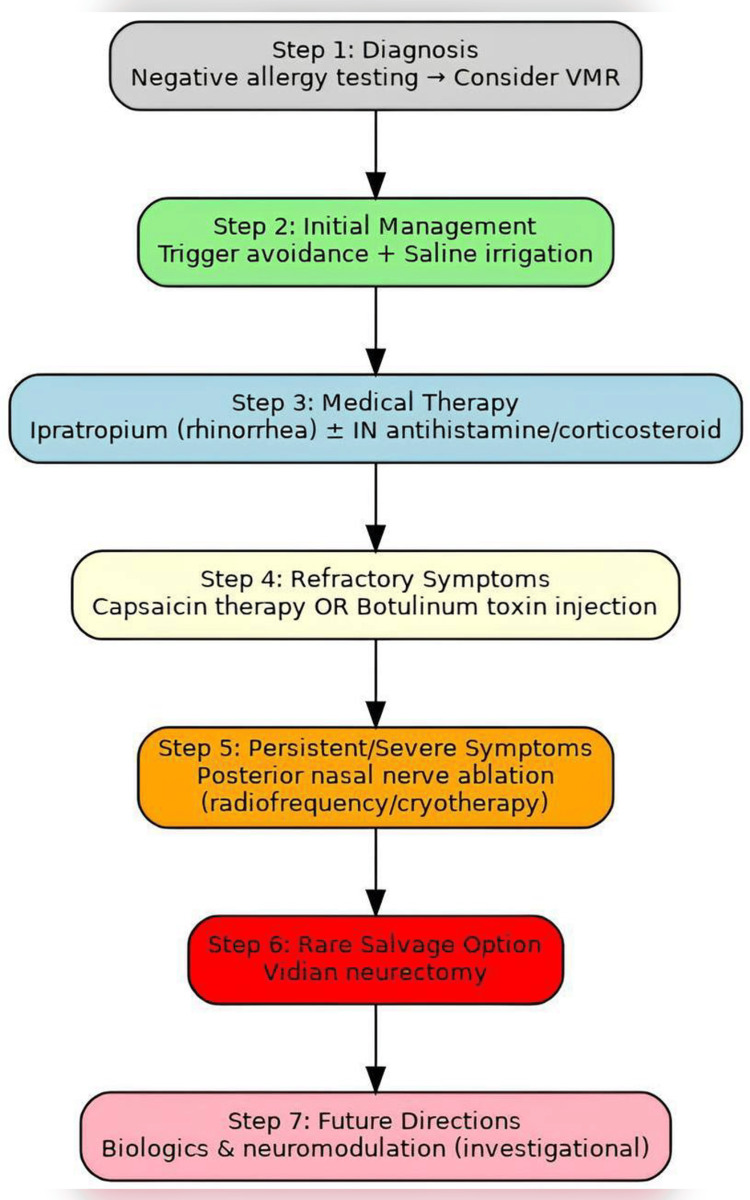
Practical treatment algorithm for vasomotor rhinitis (VMR). Stepwise approach from diagnosis to advanced therapies. Created by the authors based on current evidence [1,7,8,14,16-20].

As shown in Figure 1, the management of VMR follows a stepwise approach beginning with diagnosis and progressing to advanced procedural therapies.

Discussion

The large variation in underlying mechanisms highlights why the treatment of VMR remains challenging, as symptom responses vary widely among patients. Some patients demonstrate parasympathetic hyperactivity, whereas others show neuropeptide-mediated inflammation or heightened vulnerability to environmental stimuli [20]. This heterogeneity underscores the need for phenotype-specific therapy rather than a generalized treatment regimen. When comparing procedural and pharmacologic interventions, several key differences have been observed. Traditional sprays, including ipratropium, antihistamines, and corticosteroids, act locally and, in chronic conditions, require daily use and may be limited by side effects or incomplete symptom control [21].

By contrast, other methods, such as PNN ablation or botulinum toxin injections, offer more prolonged relief and reduced dependence on medications; however, these procedures carry potential risks and are not yet widely available in clinical practice [22]. In addition, the number of patients included in the cited studies varies considerably, reflecting differences in study design and contributing to variability in reported outcomes. Despite these advances, significant research gaps persist. There is no internationally accepted diagnostic criterion to characterize VMR, and interpretation often varies among studies, making direct comparisons difficult [21].

Procedural versus pharmacologic trials remain infrequent, and long-term outcomes beyond one to two years are poorly characterized. Moreover, most trials use heterogeneous endpoints, and few employ validated measurement tools, including minimal clinically important difference thresholds for rTNSS or SNOT-22 [23]. Therefore, future studies should aim to include larger, well-defined patient groups to ensure stronger evidence and improve the reproducibility of findings. To address these limitations, further randomized, trial-based studies with standardized outcome measures are needed to refine treatment algorithms and align them with patient-centered outcomes.

This review also has several clinical implications. The heterogeneous nature of VMR mandates shifting clinical practice toward phenotype-guided, individualized therapies. Clinicians should move beyond conventional diagnostic approaches by incorporating autonomic reflex testing or neuropeptide assays, enabling more precise patient classification and targeted therapy selection. Future research directions include developing standardized, globally accepted diagnostic criteria for VMR and conducting comparative trials between pharmacologic and procedural interventions to establish evidence-based treatment hierarchies.

Limitations

The present review has certain limitations that must be acknowledged. First, it was conducted as a narrative rather than a systematic review, which introduces potential selection and publication bias. Despite the targeted search strategy using PubMed, EMBASE, and Cochrane, language or indexing restrictions may have led to the omission of relevant studies [24]. The lack of standardized diagnostic criteria also limits comparability among studies and affects the generalizability of findings.

In addition, the number of patients included in most of the cited studies is relatively small and varies considerably between trials, which may limit the overall strength of the available evidence.

Moreover, the majority of clinical studies, as well as even the most recent ones, in this field consist of omnibus studies that are relatively small, and randomized controlled trials with long-term outcomes are rare, particularly for novel methods such as botulinum toxin, cryotherapy, and PNN ablation [25]. Such gaps underscore the need for larger multicenter studies with standardized endpoints to evaluate the sustainability, safety, and comparative effectiveness of emerging treatment modalities.

## Conclusions

VMR is a common, under-identified disorder that is neurogenic in nature. Many patients respond to oral and topical saline, topical antihistamines, corticosteroids, and ipratropium sprays as conventional treatments that relieve symptoms, and refractory responders can undergo capsaicin, botulinum toxin, and anterior nasal nerve ablation using radiofrequency or cryotherapy. The emerging therapies hold much promise.

In addition to the effect of enhanced phenotyping and quality of the clinical trials, treatment procedures may be more focused and efficient, and that, in the end, positively influences the quality of life of the affected people. It is concluded that the current review provides implications for clinical practice as well as directions for future research.

## References

[1] Leader P, Geiger Z (2025). Vasomotor Rhinitis. https://www.ncbi.nlm.nih.gov/books/NBK547704/.

[2] Lv R, Shan J, Sun A, Xing Z, Xu Q, Shao Q, Li H (2025). Research progress of anti-IGE treatment for allergic rhinitis. Am J Otolaryngol.

[3] Baroody FM, Gevaert P, Smith PK, Ziaie N, Bernstein JA (2024). Nonallergic rhinopathy: a comprehensive review of classification, diagnosis, and treatment. J Allergy Clin Immunol Pract.

[4] Meng Y, Wang C, Zhang L (2021). Diagnosis and treatment of non-allergic rhinitis: focus on immunologic mechanisms. Expert Rev Clin Immunol.

[5] Yum HY, Ha EK, Shin YH, Han MY (2021). Prevalence, comorbidities, diagnosis, and treatment of nonallergic rhinitis: real-world comparison with allergic rhinitis. Clin Exp Pediatr.

[6] Patil N, Jain S (2024). Rhinomanometry: a comprehensive review of its applications and advancements in rhinology practice. Cureus.

[7] Dykewicz MS, Wallace DV, Amrol DJ (2020). Rhinitis 2020: a practice parameter update. J Allergy Clin Immunol.

[8] Ponda P, Carr T, Rank MA, Bousquet J (2023). Nonallergic rhinitis, allergic rhinitis, and immunotherapy: advances in the last decade. J Allergy Clin Immunol Pract.

[9] Jaradeh SS, Smith TL, Torrico L, Prieto TE, Loehrl TA, Darling RJ, Toohill RJ (2000). Autonomic nervous system evaluation of patients with vasomotor rhinitis. Laryngoscope.

[10] Yao A, Wilson JA, Ball SL (2018). Autonomic nervous system dysfunction and sinonasal symptoms. Allergy Rhinol (Providence).

[11] Van Gerven L, Steelant B, Hellings PW (2018). Nasal hyperreactivity in rhinitis: a diagnostic and therapeutic challenge. Allergy.

[12] Van Gerven L, Alpizar YA, Wouters MM (2014). Capsaicin treatment reduces nasal hyperreactivity and transient receptor potential cation channel subfamily V, receptor 1 (TRPV1) overexpression in patients with idiopathic rhinitis. J Allergy Clin Immunol.

[13] Van Gerven L, Alpizar YA, Steelant B (2017). Enhanced chemosensory sensitivity in patients with idiopathic rhinitis and its reversal by nasal capsaicin treatment. J Allergy Clin Immunol.

[14] Van Gerven L, Steelant B, Alpizar YA, Talavera K, Hellings PW (2018). Therapeutic effect of capsaicin nasal treatment in patients with mixed rhinitis unresponsive to intranasal steroids. Allergy.

[15] Chang MT, Song S, Hwang PH (2020). Cryosurgical ablation for treatment of rhinitis: a prospective multicenter study. Laryngoscope.

[16] Hwang PH, Lin B, Weiss R, Atkins J, Johnson J (2017). Cryosurgical posterior nasal tissue ablation for the treatment of rhinitis. Int Forum Allergy Rhinol.

[17] Joshi SV, Rawool JS, Shaikh A, Telang RA, Sonawale S, Roy C, Thakur RT (2022). Posterior nasal nerve resection-a novel and effective way to surgically treat refractory allergic and vasomotor rhinitis. Indian J Otolaryngol Head Neck Surg.

[18] Stolovitzky JP, Ow RA, Silvers SL, Bikhazi NB, Johnson CD, Takashima M (2021). Effect of radiofrequency neurolysis on the symptoms of chronic rhinitis: a randomized controlled trial. OTO Open.

[19] Del Signore AG, Greene JB, Russell JL, Yen DM, O'Malley EM, Schlosser RJ (2022). Cryotherapy for treatment of chronic rhinitis: 3-month outcomes of a randomized, sham-controlled trial. Int Forum Allergy Rhinol.

[20] Sonoda S, Murakami D, Saito Y (2021). Long-term effectiveness, safety, and quality of life outcomes following endoscopic posterior nasal neurectomy with submucosal turbinectomy for the treatment of intractable severe chronic rhinitis. Auris Nasus Larynx.

[21] Takashima M, Stolovitzky JP, Ow RA, Silvers SL, Bikhazi NB, Johnson CD (2023). Temperature-controlled radiofrequency neurolysis for treatment of chronic rhinitis: 12-month outcomes after treatment in a randomized controlled trial. Int Forum Allergy Rhinol.

[22] Balai E, Gupta KK, Jolly K, Darr A (2023). Posterior nasal nerve neurectomy for the treatment of rhinitis: a systematic review and meta-analysis. Eur Ann Allergy Clin Immunol.

[23] Rinzin K, Hoang MP, Seresirikachorn K, Snidvongs K (2021). Botulinum toxin for chronic rhinitis: a systematic review and meta-analysis. Int Forum Allergy Rhinol.

[24] Ogi K, Valentine R, Suzuki M, Fujieda S, Psaltis AJ, Wormald PJ (2022). The anatomy of the foramina and efferent nerve fibers from the pterygopalatine ganglion in posterolateral nasal wall. Laryngoscope Investig Otolaryngol.

[25] Awan KH (2017). The therapeutic usage of botulinum toxin (Botox) in non-cosmetic head and neck conditions - an evidence based review. Saudi Pharm J.

